# Mismatch between trochlear coronal alignment of arthritic knees and currently available prosthesis: a morphological analysis of 4116 knees and 45 implant designs

**DOI:** 10.1007/s00167-022-07251-5

**Published:** 2022-12-02

**Authors:** Sergio Barroso Rosa, Kaushik Hazratwala, Matthew P. R. Wilkinson

**Affiliations:** 1 Present Address: The Orthopaedic Research Institute of Queensland, Townsville, QLD Australia; 2 James Cook University, Townsville, QLD Australia; 3 University of Tasmania, Hobart, TAS Australia; 4 Universidad de Las Palmas de Gran Canaria, Las Palmas de Gran Canaria, Canary Islands Spain

**Keywords:** Total knee replacement, Prosthesis design, Patellofemoral joint, Osteoarthritis

## Abstract

**Purpose:**

In up to a fifth of total knee replacements (TKR), surgeons are not capable of achieving good clinical and functional results. Despite comprehensive diagnostic workup, an underlying cause is not always identified in these patients. The purpose of this study is to compare native and prosthetic trochlear anatomies, to evaluate a potential source of morphologic mismatch and theoretically, of poor clinical outcomes.

**Methods:**

Native trochlear angles of 4116 knee CTs from 360 Knee Systems database of arthritic pre-operative TKR patients were evaluated. A semi-automated tridimensional analysis was performed to define the native trochlear angle in the coronal plane (NTA) among other 142 parameters. An active search was conducted to identify currently available TKR models; prosthetic trochlear orientation in the coronal plane (PTA) was extracted from the technical data provided by manufacturers.

**Results:**

The mean native trochlear angle (NTA) was 1.6° ± 6.6° (valgus) with a range from − 23.8° (varus) to 30.3°(valgus). A valgus NTA was present in 60.6% of the knees and 39.4% of them had a varus NTA. 89 TKR models were identified; trochlear details were available for 45 of them, of which 93% were designed with a valgus orientation of the prosthetic trochlear angle (PTA) and 6.9% showed a neutral (0°) PTA. Varus alignment of PTA was not present in any system. Angular numeric values for PTA were available for 34 models; these ranged from 0° to 15° of valgus, with a median value of 6.18° (SD ± 2.88°).

**Conclusion:**

This study shows a significant mismatch between native and prosthetic trochlear angles. A relevant proportion of the studied knees (41.45%) fall out of the trochlear angle range of currently available implants; representing a potential source for biomechanical imbalance. While further research is warranted to fully understand the clinical implications of the present study, manufacturers may need to take these findings into account for future implant designs.

**Level of evidence:**

Level III, retrospective cohort study.

## Introduction

Up to 20% of patients with a total knee replacement (TKR) report poor clinical outcomes [15]. Major complications are associated with suboptimal results [18]; implant malpositioning can also be a determinant factor for chronic pain and dysfunction, too [21]. Under this premise, several alignment philosophies have appeared in the last decades, but to date none of them have proved superior [4].

Moreover, patellofemoral complications are present in up to a fifth of TKRs, with underlying causes frequently remaining unidentified [24]. Initial TKR designs in the 1970s completely neglected the patellofemoral joint (PFJ) [27]; the ideal configuration of the patellofemoral component is still under debate today. It has been assumed that despite state-of-the-art procedures, patellofemoral complications will remain noticeable due to inherent limitations of available implants [5], which have shown significant tridimensional discrepancies with native trochleae [6].

TKR procedures tend to be more and more personalized [19], even though implants are mass-produced to fit average anthropometric measurements. Concerns have been raised about what should be considered as ´normal´, and whether off-the-shelf implants can be applied universally to all patients and surgical techniques: a large anatomical study showed wide variability in several tibio-femoral parameters, with a relevant proportion of arthritic knees presenting with varus-aligned trochleae [10]. As the design of the femoral groove is considered the main determinant of prosthetic patellofemoral tracking [17] and the effect of implant coronal alignment is known to have an impact on patellar loading [29]; might this native/prosthetic mismatch be one of those unidentified causes of dissatisfaction in TKR?

This piece of research has been designed to compare the trochlear coronal alignment of arthritic knees to that of currently universally available TKR models, analyzing a potential source for PFJ imbalance and subsequent poor clinical results. The authors hypothesize wide discrepancies between native and prosthetic trochleae, which may alter patellofemoral tracking and increase patellar loading of replaced knees: the clinical implication of this presumption may affect current knee replacements worldwide.

## Methods

### Data extraction

#### Native femoral anatomy

Four thousand one hundred and sixteen pre-operative knee 3DCTs (3-dimensional computed tomography) were retrospectively extracted from the 360 Knee Systems Database (Bellberry Human Research Ethics Committee, approval number 2012-03-710; Bellberry Ltd, 123 Glen Osmond Road Eastwood SA 5063 Australia). This cohort represented the entire database from January 2014 until April 2020. Participants were patients with end-stage arthritis recruited from the general Australian population, determined eligible for a primary TKR by one of the Australian 360 Knee Group surgeons. 3DCT capture and analysis was performed by *360 Knee Systems* (Pymble, NSW, Australia) with the purpose of generating pre-operative dynamic knee simulation reports that are commercially available from the company. Table 1 summarizes the general characteristics of the sample.

**Table 1:** General characteristics of the sample

Knee (side)	Left (% (*n*))	46.5% (1914)
	Right (% (*n*))	53.5% (2202)
Gender	Male (% (*n*))	44.5% (1832)
	Female (% (*n*))	55.5% (2284)
Age (mean ± SD)	71.8 ± 8.4	

Detailed 3D models were generated from CT imaging according to a standardized protocol: 3D-reconstructed femur and tibia bones are generated through semi-automated segmentation, and are used to landmark and identify points of interest by biomedical engineers using the *ScanIP* software (Simpleware, Exeter, UK). Bones are converted to stereolithography files and landmarked independently by two engineers. If any parameter differs by a threshold value, a third engineer reviews the sample. This measurement protocol showed ICC values above 0.9 in all landmarks included in a previous study, meaning excellent reliability. Maximal distance and angular measurement accuracy errors have been quantified as 0.5 mm and 0.9°, respectively [30].

**Fig. 1:**
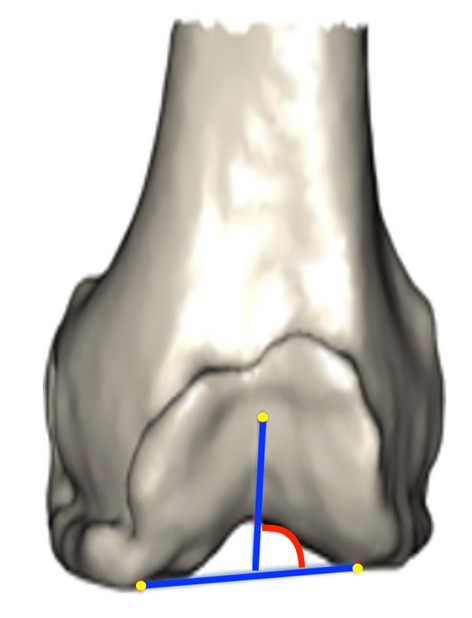
Graphic definition of native trochlear angle

For every patient, 143 morphological features were obtained. In relation to the trochlear anatomy, one measurement was taken in considerations for the purpose of the present study:

**Native trochlear angle to distal femoral angle (NTA):** the line formed by a line of best fit of the deepest valley of the trochlea sulcus to a line drawn tangential to the distal femur (Fig. 1). NTA is measured relative to the femoral coronal plane and rotationally to the anatomical transepicondylar axis.

#### Prosthetic implants

The first author (SB) conducted an active search to identify current commercially available implants for primary TKR, as of September 2022. Dedicated orthopedic publications, forums and websites, online ordering catalogues and domains from specific commercial manufacturers and distributors were examined for potential references. A list of implants was defined, including all bicondylar models found, regardless of their material, fixation technique (cemented or not) and bearing system (cruciate retaining/sacrificing, posterior stabilized, medial pivot, fixed/mobile, etc.).

**Fig. 2:**
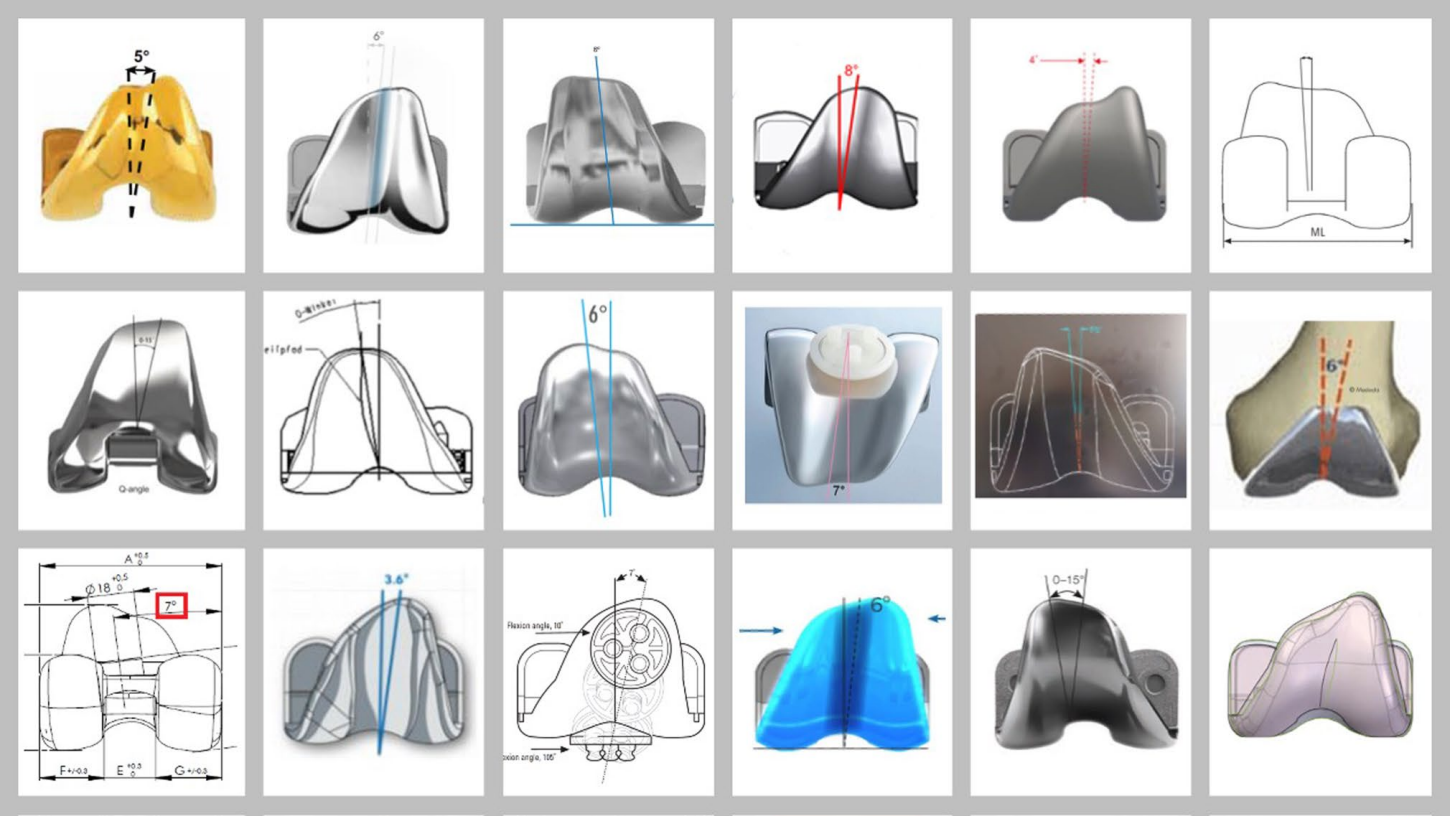
Examples of prosthetic trochlear angles in several TKR models included in this study

To obtain specific design features of femoral components, written details were acquired directly from manufacturing companies or licensed distributors. These were extracted from technical data available in the company’s websites/catalogues, or by means of direct request to their technical/scientific departments (Fig. 2). Overall orientation (valgus, varus, or neutral) in relation to the component coronal plane (i.e., perpendicular to the joint line/distal condylar angle) and numeric value of the prosthetic trochlear angle were recorded when available.

**Fig. 3:**
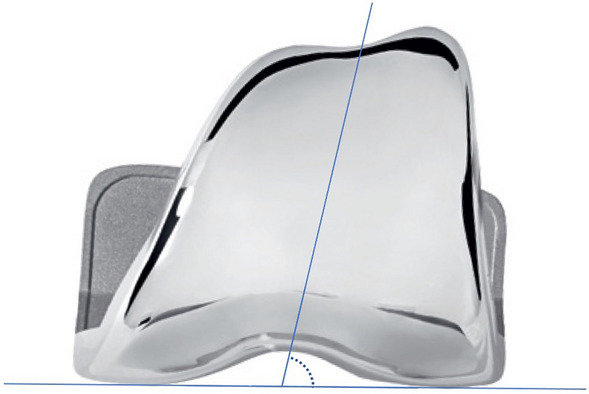
Definition of PTA

**Prosthetic trochlear angle (PTA):** defined as the angle resulting from bisecting the prosthetic distal condylar line and the trochlear sulcus direction in the coronal plane (Fig. 3).

### Data analysis

Statistical analysis was performed in IBM SPSS Statistics 27 (IBM Corp. Released 2020. IBM SPSS Statistics for Windows, Version 27.0. Armonk, NY: IBM Corp). The Kolmogorov–Smirnov test was employed to determine sample normality. The Mann–Whitney *U* test was selected to compare sample means (nonparametric). Categorical variables were summarized using percentages and relative frequencies. Numerical variables were summarized by the mean ± standard deviation (SD).

## Results

### Native trochlear anatomy

The mean NTA was 1.6° ± 6.6° (valgus) with a range of values from − 23.8° (varus) to 30.3°(valgus). NTA normal distribution resulted in 60.6% of the knees showing a valgus alignment, while 39.4% of the knees showed varus values (Table 2). Among the 4116 knees, 220 specimens (5.3%) had a ‘virtually neutral’ NTA, i.e., an angulation between 0.5° of varus and 0.5° of valgus.

**Table 2:** Descriptive of native and prosthetic trochlear angles

	Native—NTA	Prosthetic—PTA	*p* value
Mean angle*	1.6°(± 6.6°)	6.18° (± 2.88°)	0.00003
Range	− 23.8° to 30.3°	0°–15°	
Distribution**			0.0003
Valgus	60.6% (5.7° ± 4.5°)	91.9% (5.96° ± 1.7°)	
Varus	39.4% (− 4.7° ± 3.8°)	0%	
Neutral (0°)	0%	8.9%	

* Shown as averages (± SD). ** Shown as percentage (average ± SD). Negative values = varus. Positive values = valgus

### Prosthetic trochlear anatomy

For primary knee replacement, 89 available bicondylar implants were identified. These correspond to 60 manufacturers from 17 countries in Europe, North and South America, Asia, and Oceania. Trochlear design data were available for 45 brands (50.5% of the identified models) (Table 3).

**Table 3:** List of implants, sorted by alphabetical order

Implant name	Manufacturer	Country	Trochlear orientation	Trochlear angle	Observations
4-motion	Artiqo	Germany	Valgus	9°	
4 Fit	K implant	Germany	Valgus	5°	
ACS	Implantcast	Germany	Valgus	5°	
Anatomic	Amplitude	France	Valgus	6°	
Apex	Corin	UK	Valgus	6°	
Attune	Depuy-Syntes	USA	Valgus	10°–14°	Variable PTA according to implant size
Balansys	Mathys	Switzerland	Valgus	–	
Bone01	Walkman	China	Valgus	7°	
Cinetique	Medacta	Switzerland	Valgus	6°	
Columbus	Braun	Germany	Valgus	7°	
Consensus Knee	Consensus Orhopaedics	USA	Valgus	6°	
Cygnus	Walkman	China	Valgus	7°	
Cynthia	Double Medical	China	Valgus	–	
Empower	DJO Surgical	USA	Valgus	–	
Evolution	Microport	USA	Valgus	3.6°	
Exult	Corentec	South Korea	Valgus	–	
Freedom	Meril	India	Valgus	6°	
Future	Biotech Medical	Germany	Valgus	7°	
Gemini	Link	Germany	Valgus	6°–8.3°	Variable PTA according to implant size
Genesis II	Smith and Nephew	UK	Valgus	*	* S-shaped trochlea
Genus	Adler Ortho	Italy	Valgus	8°	
Genutech	Surgival	Spain	Valgus	6°	
GMK	Medacta	Switzerland	Valgus	6°	
High Flex	Biotech Medical	Germany	Valgus	7°	
Journey II	Smith and Nephew	UK	Valgus	*	* S-shaped trochlea
K-MOD	Bioimpianti	Italy	Valgus	6°	
LCS	Depuy-Syntes	USA	Neutral	0°	
Legion Primary	Smith and Nephew	UK	Valgus	*	* S-shaped trochlea
Logic	Exactech	USA	Neutral	0°	Variable tracking 9° to -9°
Lospa	Corentec	South Korea	Valgus	–	
MRK	Mathorto	UK	Neutral	0°	
Nexgen	Zimmer-Biomet	USA	Valgus	7°	
Persona	Zimmer-Biomet	USA	Valgus	7°	
Saiph	Mathorto	UK	Neutral	0°	
Score	Amplitude	France	Valgus	6°	
Score 2	Amplitude	France	Valgus	6°	
Sigma	Depuy-Syntes	USA	Valgus	11.9°–13.3°	Variable PTA according to implant size
SKI	Just Medical	China	Valgus	15°	
SKS	Aston Sem	France	Valgus	–	
Triathlon	Stryker	USA	Valgus	–	
TP	Biotech Medial	Germany	Valgus	7°	
U2	United Orthopedics	Taiwan	Valgus	4°	
Unity	Corin	UK	Valgus	–	
Vanguard	Zimmer-Biomet	USA	Valgus	6.5°	
XN	Chun-Li	China	Valgus	7°	

From this list of 45 designs, a majority (91.1%) showed a valgus orientation of the PTA, while four implants (8.9%) were designed with a neutral (0°) PTA. PTA varus alignment was not present in any system. Angular numeric values for PTA were available for 34 models, including the 10 most implanted TKR models in Australia in the 2014–2021 period, accounting for the 67.2% of TKRs implanted nationwide [1]. These ranged from 0° to 15° (neutral to valgus), with a mean value of 6.18° (Table 2).

## Discussion

**Fig. 4:**
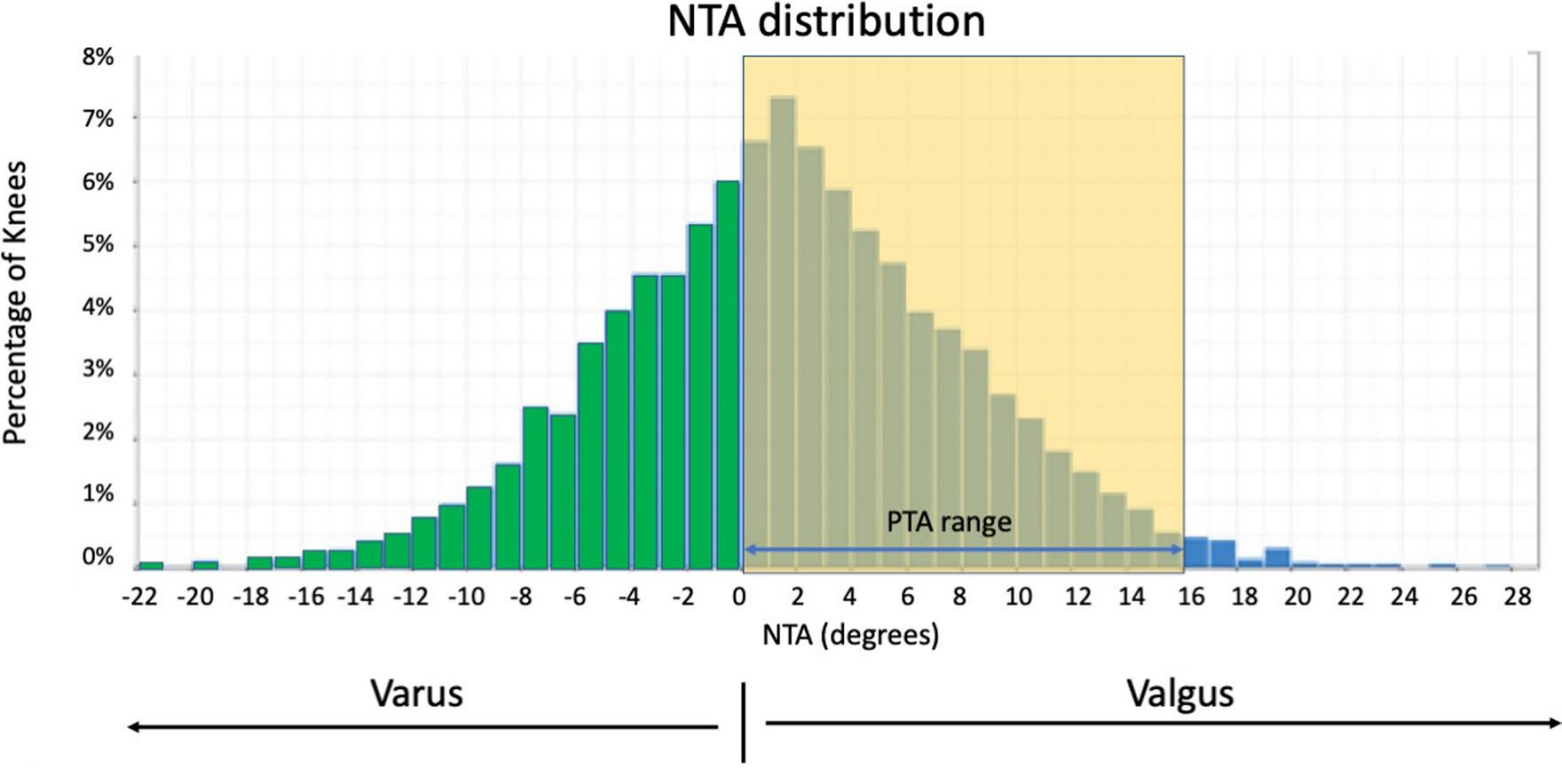
Distribution of native trochlear angles (NTA) in the study cohort. Green bars represent varus alignment, while blue bars correspond to valgus alignment. Shaded area reflects the prosthetic trochlear angle (PTA) range

The main finding of this study is that trochlear coronal alignment of arthritic knees and that of current TKR implants differ significantly (Table 2). According to the analysis, only 58.55% of knees in the sample would fall in a matched PTA-NTA range if all the studied models were available for implantation, using the mechanical alignment technique (Fig. 4). With a non-mechanical technique, the PTA-NTA would further change; typically, positioning the femoral component in valgus will orientate the prosthetic trochlea in varus (and vice versa), increasing the NTA–PTA discrepancy (as 60.6% of knees have a valgus NTA). It is noteworthy that two-thirds of all primary knee prostheses implanted in Australia in 2014–2021 have been included in this analysis [1], which emphasizes the potential significance of this mismatch in the clinical scenario.

Dejour et al*.* conducted a morphologic analysis of trochlear design in 14 TKR models available in 2014, with similar results. All trochleae were valgus-oriented (mostly in the 5°–8° range) except for one, which was neutral [5]. Therefore, at least in regards to trochlear coronal orientation, there has been little evolution during the last decade. The trochlear design of current TKR models is based on the assumption that most native knees present a valgus trochlea [2]; this concurs with the present results showing a mean PTA of 6.18° of valgus. However, the analysis also suggests that NTA range is more varus (or less valgus) than it has been generally considered [10].

There are a handful of previous reports rejecting valgus alignment as the natural conformation of the femoral trochlea. Ekhoff et al. and Koh et al*.* described a neutral orientation of the femoral trochlea, with means of 0.4° ± 5° and − 0.1° ± 9°, respectively [8] [16]. The cadaveric study by Barik et al*.* revealed an average varus orientation (1.8° ± 2.1°) of the femoral groove [3], while Grassi et al*.* also encountered 20% of varus-aligned trochleae in a navigation study with 110 arthritic knees [9]. In the present sample, which is by far the largest published, the mean value was valgus (1.6° ± 6.6°), but 39.4% of knees had a varus-oriented trochlea. Therefore, the consideration of a laterally oriented femoral trochlea as a standard feature of the femoral component should be approached with caution. High variability has been observed in the axial plane morphology of arthritic trochleae, too [12].

Riviere et al. reported a high degree of discrepancy between native and prosthetic trochlear alignment in kinematically aligned TKR, with a more valgus orientation of the latter [25]. In additional studies, this discrepancy was also shown for mechanical alignment [13, 26]; this could be interpreted as an inherent limitation of implant designs. Barink has already proposed a more truly anatomical femoral configuration, with a more medially oriented trochlea [2]. In fact, one of the implants with a neutral (0°) PTA showed good patellofemoral performance, with just 5.6% of residual anterior knee pain in a cohort of 1482 TKRs [7]. In addition, according to manufacturers, several models have a ‘*widened trochlear angle*’, in theory enabling the accommodation of a range of NTAs within a limited extent. Further analysis is warranted to determine the biomechanical and clinical impact of this concept. Besides that, the inception of patient-specific implants may represent a genuine solution to accommodate the wide variability of trochlear [20] and other knee parameters [22]. Longer follow-ups are required to evaluate clinical superiority [31], and a significant reduction in current costs is also required to permit more widespread use. Ultimately, manufacturing companies may need to take this wide variability into account, finding ways to accommodate outlying anatomies, and probably, offering a wider range of prosthetic trochlear orientations.

Positioning of the femoral component with a mild external rotation (3° according to Insall) has been a general recommendation to favor adequate patellar tracking in TKR [23]. However, a previous publication revealed that native trochleae were internally rotated in 27.1% of the cases [10]: external rotation of the femoral implant may exaggerate the NTA–PTA discrepancy. Moreover, it has been analyzed how femoral component rotation critically affects tibial rotation, ligament forces, retropatellar stress, and varus–valgus position [32], highlighting the narrow margin a surgeon has for balancing patellar tracking only by adjusting this parameter.

The results of this study suggest the NTA–PTA mismatch as a feasible cause for biomechanical imbalance and dysfunction. Hochereiter et al*.* concluded that “*any valgisation in TKA will increase (…) lateral PFJ contact pressure*” [11]; the present article highlights that PTA is indeed more valgus than native values. This mismatch can be only identified preoperatively by means of 3D CT analysis, allowing surgeons to anticipate a surgical planning to accommodate wide discrepancies. Navigation can also be of extreme usefulness, even if 3D CT is lacking. However, these routines are still not a standard procedure in many settings; a 2021 review revealed that the proportion of navigated TKR was only 32% in Australia (2019), 30% in Germany (2014), 6.3% in USA (2014), and 3% in the UK (2014) [28]; not to mention less wealthy regions. In consequence, substantial variations in NTA may be left unappreciated, potentially compromising current clinical outcomes.

This study has some limitations. Native data have been extracted from arthritic knees; it could be argued that the results are not a reflection of undamaged knees. This would occur only after some degree of bone loss due to advanced disease being present; patients in Australia generally present at early stages of OA, and the sample size would probably eliminate this theoretical deviation. Notwithstanding that, these knees are the ones requiring a replacement, and therefore, the comparison of such anatomical features to those of implants in use has been considered adequate. Either way, a previous study on healthy knees revealed a trochlear valgus orientation of 1° ± 5° [14], a result equally distant from the average PTA values. Another limitation was the inability to source PTA details of all implants currently on the market. Dejour et al*.* already sentenced that manufactures tend to provide scarce details in regard to trochlear parameters [5]. Nonetheless, it appears unlikely to have missed models with significantly different PTAs, as this would probably have been advertised as a genuine feature in the brochures available for all systems. Further, the authors acknowledge that there are other trochlear parameters such as congruity, depth, lateral height, and the patellar component shape itself that can influence balance, tracking, and functional outcome. Finally, the clinical correlation of patellofemoral complications and trochlear mismatch within the studied cohort has not been analyzed. The fact that this cohort encompasses the records of multiple surgeons across Australia, employing varied surgical protocols and implants, has hindered this analysis, which remains as a future objective. As a consequence, the clinical implications of the NTA–PTA incongruity are still to be defined.

## Conclusion

Trochlear coronal orientation in arthritic knees and in currently used implants differ relevantly, which may contribute to patellofemoral imbalance and dissatisfaction after TKR surgery. This mismatch may be especially relevant in newer alignment techniques, where femoral components may be implanted in valgus and/or in internal rotation, further increasing the NTA–PTA discrepancy. An increased awareness is recommended, encouraging surgeons to preoperatively identify discrepancies between the native and implanted trochlea.

## Data Availability

The datasets generated during and/or analysed during the current study are available from the corresponding author on reasonable request.

## Abbreviations

TKR: Total knee replacement
OA: Osteoarthritis
CT: Computed tomography
PFJ: Patellofemoral joint
NSW: New South Wales
3D: 3 Dimensions
NTA: Native trochlear angle
PTA: Prosthetic trochlear angle
